# Kinetic estimated glomerular filtration rate in critically ill patients: beyond the acute kidney injury severity classification system

**DOI:** 10.1186/s13054-017-1873-0

**Published:** 2017-11-18

**Authors:** Flávio de Oliveira Marques, Saulo Aires Oliveira, Priscila Ferreira de Lima e Souza, Wandervânia Gomes Nojoza, Maiara da Silva Sena, Taynara Muniz Ferreira, Bruno Gabriele Costa, Alexandre Braga Libório

**Affiliations:** 1 Medical Sciences Postgraduate Program, Department of Clinical Medicine, Universidade Federal do Ceará, Fortaleza, Ceará Brazil; 2 Medical Sciences Postgraduate Program, Universidade de Fortaleza – UNIFOR, Fortaleza, Ceará Brazil; 3 Instituto Dr. José Frota, Avenida Abolição, 4043 Ap 1203, Fortaleza, Ceará CEP 60165-082 Brazil

## Abstract

**Background:**

Although significant advances have been achieved in acute kidney injury (AKI) research following its classification, potential pitfalls can be identified in clinical practice. The nonsteady-state (kinetic) estimated glomerular filtration rate (KeGFR) could add clinical and prognostic information in critically ill patients beyond the current AKI classification system.

**Methods:**

This was a retrospective analysis using data from the Multiparameter Intelligent Monitoring in Intensive Care II project. The KeGFR was calculated during the first 7 days of intensive care unit (ICU) stay in 13,284 patients and was correlated with outcomes.

**Results:**

In general, there was not a good agreement between AKI severity and the worst achieved KeGFR. The stepwise reduction in the worst achieved KeGFR conferred an incremental risk of death, rising from 7.0% (KeGFR > 70 ml/min/1.73 m^2^) to 27.8% (KeGFR < 30 ml/min/1.73 m^2^). This stepwise increment in mortality remained in each AKI severity stage. For example, patients with AKI stage 3 who maintained KeGFR had a mortality rate of 16.5%, close to those patients with KeGFR < 30 ml/min/1.73 m^2^ but no AKI; otherwise, mortality increased to 40% when both AKI stage 3 and KeGFR < 30 ml/min/1.73 m^2^ were present. In relation to another outcome—renal replacement therapy (RRT)—patients with the worst achieved KeGFR < 30 ml/min/1.73 m^2^ and KDIGO stage 1/2 had a rate of RRT of less than 10%. However, this rate was 44% when both AKI stage 3 and a worst KeGFR < 30 ml/min/1.73 m^2^ were observed. This interaction between AKI and KeGFR was also present when looking at long-term survival.

**Conclusion:**

Both the AKI classification system and KeGFR are complementary to each other. Assessing both AKI stage and KeGFR can help to identify patients at different risk levels in clinical practice.

**Electronic supplementary material:**

The online version of this article (doi:10.1186/s13054-017-1873-0) contains supplementary material, which is available to authorized users.

## Background

Acute kidney injury (AKI) is now recognized as a major public health problem affecting millions of patients worldwide [1]. Critically ill patients are at high risk of developing AKI, with its incidence during intensive care unit (ICU) stay varying from 36% to 67% [2, 3]. During the last few years, acute-onset disturbance of kidney function has been a subject of avid scientific discussion, which has led to the definition of “acute kidney injury.” AKI identification was based on changes in serum creatinine (SCr) compared with baseline levels before the disease onset and changes in diuresis. Scoring systems for AKI quantification have been developed at consensus conferences. These included the RIFLE [4] and AKIN [5] criteria for AKI. Most recently, the AKIN criteria were revised and clarified as the Kidney Disease Improving Global Outcomes (KDIGO) criteria for AKI [6].

Although significant advances have been achieved in AKI research following this classification, potential pitfalls can be identified in clinical practice. Intuitively, the shorter the amount of time during which a determined SCr change occurs, the greater the AKI severity. For example, going from a SCr of 1 to 1.5 mg/dl within 12 h signifies a worse glomerular filtration rate (GFR) fall than going from a SCr of 1 to 1.5 mg/dl within 48 h (see Additional file 1 for illustrative examples); however, if the same urinary output is maintained in both situations, AKI severity will be classified similarly. Also, even considering the difficulty in ascertaining a baseline SCr, the AKI score systems do not consider previous underlying chronic kidney disease (CKD) and its possible prognostic implications. To exemplify, a patient whose SCr varied from 0.8 to 1.2 mg/dl has the same AKI severity as another patient whose variation was from 2 to 3 mg/dl, although the GFR is clearly more severely reduced in the second case. Finally, as suggested by Waikar and Bonventre [7] and demonstrated by our group [8], an SCr kinetic model can be superior to AKI classification systems in patients with previous CKD.

Assessing the GFR is problematic when the SCr is changing quickly. In severe AKI and anuric patients, it is a consensus to consider that the GFR is < 10 ml/min/1.73 m^2^. However, a less reduced GFR may also affect management and impact patient survival. Recently, the nonsteady-state (kinetic) estimated glomerular filtration rate (KeGFR) has been advocated in AKI and renal recovery assessment [9, 10]. The formula is derived from the initial SCr, the distribution volume, the creatinine production rate, and the quantitative difference between consecutive SCr over a given period. Taking these variables into account, KeGFR yields the measured creatinine clearance (CrCl) rate for that period between two SCr measurements. Thus, the KeGFR results in the same interpretation of a measured CrCl level, but without the need for collecting urine and measuring urinary creatinine levels. Using this approach, we can estimate the GFR in a determined time interval, regardless of whether Scr is slowly increasing as described in the abovementioned examples, where a patient whose SCr levels increased from 1 to 1.5 mg/dl in 12 h had a worse KeGFR in comparison with another whose SCr level also increased from 1 to 1.5 mg/dl in 48 h.

In the present study, we hypothesized that a worse KeGFR could add clinical and prognostic information in critically ill patients beyond the current AKI classification system, mainly regarding the need for renal replacement therapy, hospital mortality, and 1-year survival.

## Methods

### Multiparameter Intelligent Monitoring in Intensive Care II database and data collection

The Multiparameter Intelligent Monitoring in Intensive Care (MIMIC)-II project, maintained by the Massachusetts Institute of Technology Laboratory for Computational Physiology, contains data on patients hospitalized in an ICU at Beth Israel Deaconess Medical Center from 2001 to 2008 [11]. The database is freely available so that any researcher who accepts the data use agreement and has attended “protecting human subjects’ training” can apply for permission to access the data. This study was approved by the institutional review boards of Massachusetts Institute of Technology and Beth Israel Deaconess Medical Center and was granted a waiver of informed consent.

We included all patients with an ICU length of stay (LOS) lasting more than 48 h with at least three SCr measurements taken. Patients with known end-stage renal disease (ESRD), previous renal transplantation, those who underwent renal replacement therapy (RRT) before ICU admission, and those with admission SCr > 4 mg/dl were excluded.

### Data collection

All data were extracted from the MIMIC-II database (v2.6) and included demographic information (e.g., age, gender) and clinical information from the admission notes. The following admission data were collected: admission body weight, admission type (elective or emergency), care unit type (medical, coronary unit, surgery, or cardiac surgery), sepsis diagnosis as described by Angus et al. [12], admission SCr, and disease severity as assessed by the Simplified Acute Physiology Score (SAPS) II [13] and Sequential Organ Failure Assessment (SOFA) [14] scores. In the first 7 days of ICU stay, we also recorded daily SCr measurements, and the need for vasoactive drugs and mechanical ventilation.

### Estimated kinetic glomerular filtration rate

The KeGFR was calculated during the first 7 days of ICU stay according the following equation:

$$ KeGFR=\frac{baseline\; SCr\;X\; eGFR}{Mean\; SCr} $$

$$ x\left[1\hbox{-} \frac{24\mathit{\mathsf{x}}\varDelta \mathsf{SCr}}{\varDelta Time(h)\mathit{\mathsf{x}} Ma\mathit{\mathsf{x}}\varDelta SCr/ day}\right] $$

where eGFR = estimated glomerular filtration rate using baseline SCr, mean SCr = mean of two consecutive SCr measurements, ΔSCr = change in SCr, ΔTime(h) = interval in hours between two consecutive SCr measurements, and MaxΔSCr/Day = the maximal change (increase) in SCr that can occur per day if renal function is completely lost.

The KeGFR was derived from the initial SCr, the distribution volume, the creatinine production rate, and the quantitative difference between consecutive SCr measurements over a given time. We included all SCr levels measured at least 6 h and no more than 48 h apart. KeGFR was calculated by taking each interval between two consecutive creatinine measurements. The volume of distribution for creatinine does not need to be equated with total body water, but can be expressed as a function of the creatinine production rate. The amount by which a known creatinine production rate can increase the creatinine concentration if all excretion has ceased (i.e., near-zero GFR) informs us about the volume of distribution. Since there is only creatinine addition and no subtraction, this situation describes the maximum increment of SCr in 1 day. To obtain it, we identified 94 patients with anuria and two SCr measurements apart with no RRT in this interval. The mean SCr incremental corrected for 24 h was 1.47 ± 0.44 mg/dl for men and 1.41 ± 0.49 mg/dl for women. We used these values instead of a fixed value of 1.7 mg/dl per day, as described by Chen [9].The other necessary variables for this formula included baseline SCr. Because the MIMIC-II does not provide any laboratory information prior to ICU admission, the lowest SCr available during ICU stay before RRT initiation was used as the baseline renal function and baseline CrCl was calculated using the CKD-EPI formula [15]. Any SCr measurement after RRT was not considered. After that, to exclude any influence from previous CKD or out-of-ICU-acquired AKI, we performed a complete sensitivity analysis using only patients admitted to the ICU with an eGFR > 70 ml/min/1.73 m^2^.

### AKI definition

AKI was defined according to the KDIGO criteria [6]. We classified patients based on the KDIGO maximum stage achieved within the first 7 days of ICU stay. Because we used RRT as an outcome, we did not apply it as a rule to patients that commenced RRT before achieving AKI stage 3. Urinary output was collected in fixed blocks of 6 h beginning at ICU admission. To be acceptable, the maximum gap between two actual values was 3 h. To stage a patient based on urine output (UO), a minimum of 6 h of data were required. Since Kellum et al. [16] have recently described that the risk of death over the index hospital stay and over the following year is greatest for patients that meet both UO and SCr criteria for AKI, and to exclude the fact that KeGFR is the only other approach to describe SCr increment, we performed a second sensitivity analysis with SCr-based KDIGO criteria only.

### Estimated GFR using steady-state SCr formula

Although KeGFR theoretically provides better estimation of GFR than formulae that were developed to be used with steady-state SCr, we evaluated the capacity of the most recently proposed formula (CKD-EPI) [15] using the highest SCr during the first 7 days of ICU stay to predict the main outcomes.

### Outcomes

Patient outcomes included the need for RRT during ICU stay, hospital mortality, and survival up to 1 year.

### Statistical analysis

Patients were categorized in groups according to the worst achieved KeGFR. Variables were assessed for normality using the Kolmogorov-Smirnov test. Parametric variables were compared using a *t* test and nonparametric variables using the Mann-Whitney test. Categorical variables were compared using the chi-square test. We built a logistic regression model to assess the association between categorized KeGFR and hospital mortality according to each AKI KDIGO stage. We defined a priori that the following variables would be included in the logistic regression model for both outcomes: age, gender, SAPS II score, SOFA score, main comorbidities (hypertension, congestive heart failure, cardiac arrhythmias, chronic pulmonary obstructive disease, diabetes mellitus, lymphoma, metastatic cancer, liver disease, obesity), type of admission (clinical or surgical), vasoactive drugs, the need for mechanical ventilation, and baseline eGFR. A Cox model was performed to access survival by AKI severity and lowest KeGFR after adjusting for comorbidities, baseline eGFR, and age.

## Results

### Characteristics of study patients

**Fig. 1:**
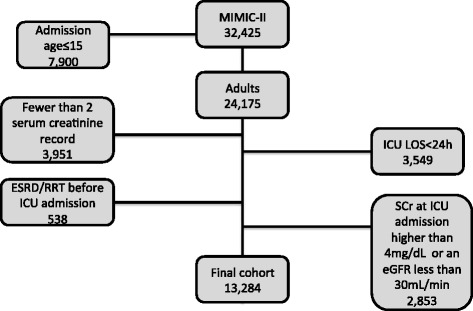
Patient distribution in the MIMIC-II database and exclusion criteria. *eGFR* estimated glomerular filtration rate, *ESRD* end-stage renal disease, *ICU* intensive care unit, *LOS* length of stay, *MIMIC* Multiparameter Intelligent Monitoring in Intensive Care, *RRT* renal replacement therapy, *sCr* serum creatinine

The MIMIC-II database contains the records of 32,425 patients, 24,175 of whom were adults aged > 15 years at the time of admission. Patients with an ICU LOS < 24 hours (*n* = 3549), those with less than two SCr measurements in a period of 48 h (*n* = 3951), and patients with ESRD/RRT before the ICU admission (*n* = 538) were excluded from the analysis. Additionally, patients at ICU admission with an SCr higher than 4 mg/dl (*n* = 1159) or an eGFR lower than 30 ml/min/1.73 m^2^ (*n* = 1694) were also excluded (Fig. 1). After all exclusions, 13,284 patients were eligible to be analyzed. Of the SCr levels used to calculate KeGFR, more than 85% had an interval between them of 20 to 28 h. Table 1 provides baseline characteristics of patients according to the worst KeGFR. Patients with a reduced KeGFR during the first 7 days of ICU stay were more prone to have more comorbidities (except arterial hypertension), more sepsis diagnosis, and were more severely ill.

**Table 1:** Baseline characteristics for patients according to worst KeGFR in the first 7 days of ICU stay

	Total patients (*n* = 13,284)	Lowest KeGFR > 70 ml/min/1.73 m^2^ (*n* = 7,089)	Lowest KeGFR 45–70 ml/min/1.73 m^2^ (*n* = 3,420)	Lowest KeGFR 30–45 ml/min/1.73 m^2^ (*n* = 1,112)	Lowest KeGFR < 30 ml/min/1.73 m^2^ (*n* = 1,663)	*p*
Age (years), mean ± SD	63.3 ± 17.3	60.3 ± 17.3	66.8 ± 16.6	69.5 ± 15.2	64.6 ± 17.0	<0.001
Male, *n* (%)	7067 (53.2)	3850 (54.3)	1772 (51.8)	596 (53.6)	849 (51.0)	0.196
Hypertension, *n* (%)	4076 (30.7)	2349 (33.1)	1073 (31.4)	311 (28.0)	343 (20.6)	<0.001
Uncomplicated diabetes, *n* (%)	2477 (18.6)	1294 (18.2)	676 (19.8)	238 (21.4)	269 (16.2)	0.001
Complicated diabetes, *n* (%)	604 (4.5)	175 (2.5)	208 (6.1)	69 (6.2)	152 (9.1)	<0.001
Obesity, *n* (%)	205 (1.5)	102 (1.4)	54 (1.6)	16 (1.4)	32 (1.9)	0.084
Congestive heart failure, *n* (%)	2566 (19.3)	1051 (14.8)	762 (22.3)	289 (26.0)	464 (27.9)	<0.001
COPD, *n* (%)	2054 (15.5)	1045 (14.7)	567 (16.6)	190 (17.1)	252 (15.1)	0.087
Cardiac arrhythmia, *n* (%)	2414 (18.2)	1180 (16.6)	672 (19.6)	224 (20.1)	338 (20.3)	<0.001
Liver disease, *n* (%)	727 (5.5)	334(4.7)	155(4.5)	82 (7.4)	156 (9.4)	<0.001
Metastatic cancer, *n* (%)	620 (4.7)	333 (4.7)	167 (4.9)	39 (3.5)	81 (4.9)	0.197
Surgical patients, *n* (%)	5275 (39.7)	2746 (38.7)	1512 (44.2)	471 (42.4)	546 (32.8)	<0.001
Sepsis, *n* (%)	3964 (29.8)	1666 (23.5)	1084 (31.7)	415 (37.3)	799 (48.0)	<0.001
SAPS II on ICU admission, median (IQR)	33 (24–41)	29 (22–38)	34 (27–43)	38 (30–47)	40 (31–49)	<0.001
SOFA on ICU admission, median (IQR)	6 (3–9)	5 (3–8)	7 (3–9)	8 (4–11)	9 (5–12)	<0.001
Baseline eGFR* (ml/min), mean ± SD	109.0 ± 33.9	129.0 ± 38.8	97.5 ± 29.9	80.9 ± 26.0	64.4 ± 34.7	<0.001
Vasopressors, *n* (%)	4793 (36.1)	2035 (28.7)	1377 (40.3)	539 (48.5)	842 (50.6)	<0.001
Mechanical ventilation, *n* (%)	8484 (63.9)	4022 (56.7)	2359 (69.0)	837 (75.3)	1266 (76.1)	<0.001
Lowest KeGFR during first 7 days of ICU stay (ml/min), mean ± SD	66.4 ± 29.1	88.2 ± 15.7	56.3 ± 7.2	34.5 ± 4.3	15.6 ± 8.1	<0.001
ICU LOS (days), median (IQR)	3.3 (2.1–6.1)	2.8 (1.8–4.8)	3.7 (2.3–6.9)	4.3 (2.6–7.7)	5.3(3.1–10.2)	<0.001
Hospital LOS (days), median (IQR)	9 (6–15)	8 (4–13)	9 (6–16)	11 (7–18)	12 (8–21)	<0.001
ICU mortality, *n* (%)	1,023 (7.7)	275 (3.9)	253 (7.4)	134 (12.0)	361 (22.7)	<0.001
Inhospital mortality, *n* (%)	1,562 (11.8)	499 (7.0)	420 (12.2)	180 (16.2)	463 (27.8)	<0.001

*Using lowest serum creatinine during ICU stay

*COPD* chronic obstructive pulmonary disease, *eGFR* estimated glomerular filtration rate, *ICU* intensive care unit, *IQR* interquartile range, *KeGFR* kinetic estimated glomerular filtration rate, *LOS* length of stay, *SAPS II* Simplified Acute Physiology Score II, *SD* standard deviation, *SOFA* Sequential Organ Failure Assessment

### Main outcomes according to the worst KeGFR

To explore the association between reduced KeGFR and observed outcomes, we first categorized the worst KeGFR achieved in the first 7 days. The cut-off values (30, 45, and 70 ml/min/1.73 m^2^) were chosen to maintain the best discriminatory capacity for hospital mortality in comparison with the worst KeGFR as a continuous variable (Additional file 2: Figure S1). As shown in Table 1, a stepwise reduction in the worst achieved KeGFR conferred an incremental risk of death, rising from 7.0% (KeGFR > 70 ml/min/1.73 m^2^) to 27.8% (KeGFR < 30 ml/min/1.73 m^2^). This association was also observed in the other evaluated outcomes (need for RRT, ICU LOS, and hospital LOS) (Table 1).

### Association between the worst achieved KeGFR and AKI stage

The distribution of patients according to AKI stage and the worst KeGFR are shown in Table 2. There was not a good agreement between AKI severity and the worst achieved KeGFR. We identified many patients with AKI according to the KDIGO classification (stages 1–3) but who maintained a KeGFR above 70 ml/min/1.73 m^2^. Although many of these patients achieved maximum KDIGO stage only by the UO criteria, 1008 patients had AKI according to the SCr-based KDIGO classification but no significant reduction in KeGFR (Additional file 3: Table S1). On the other hand, several patients achieved a KeGFR < 45 ml/min/1.73 m^2^ but had no AKI or had KDIGO AKI stage 1 only. As shown in Table 3, which includes only patients with ICU admission eGFR > 70 ml/min/1.73 m^2^, this finding cannot be explained only by previous CKD.

**Table 2:**
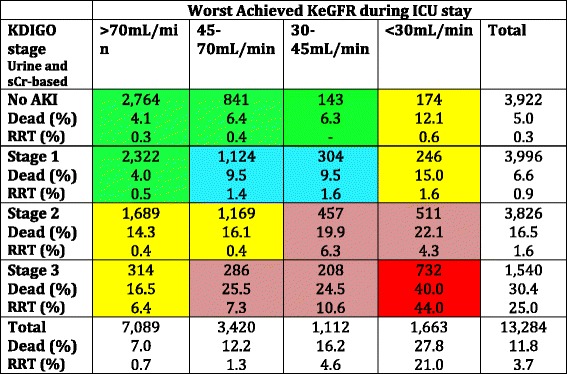
Outcomes for all patients according to maximum AKI severity and worst achieved KeGFR

Colors denote similar outcome patterns

*AKI* acute kidney injury, *ICU* intensive care unit, *KDIGO* Kidney Disease Improving Global Outcomes, *KeGFR* kinetic estimated glomerular filtration rate, *RRT* renal replacement therapy, *SCr* serum creatinine

**Table 3:**
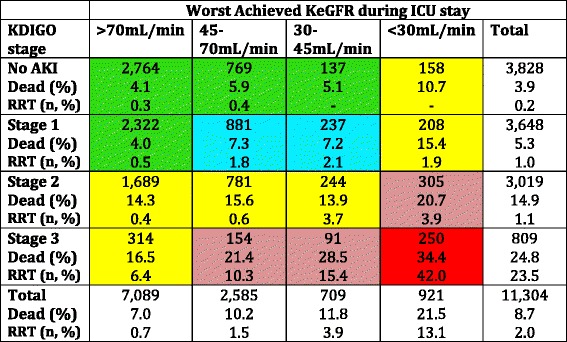
Outcomes for patients with baseline eGFR > 70 ml/min/1.73 m^2^ according to maximum AKI severity and worst achieved KeGFR

Colors denote similar outcome patterns

*AKI* acute kidney injury, *eGFR* estimated glomerular filtration rate, *ICU* intensive care unit, *KDIGO* Kidney Disease Improving Global Outcomes, *KeGFR* kinetic estimated glomerular filtration rate, *RRT* renal replacement therapy, *SCr* serum creatinine

### Impact of the worst achieved KeGFR on hospital mortality

As previously stated, the hospital mortality rate increases according to the worst achieved KeGFR within the first 7 ICU days. This stepwise increment in mortality remained in each AKI severity stage (Table 2). Roughly, it is also possible to identify an increment in hospital death when using the KDIGO system within each worse KeGFR range. For example, patients with AKI stage 3 who maintained KeGFR had a mortality rate of 16.5%, close to those patients with KeGFR < 30 ml/min/1.73 m^2^ but no AKI; otherwise, mortality increased to 40% when both AKI stage 3 and KeGFR < 30 ml/min/1.73 m^2^ were present. For illustrative purposes, we reduced the number of groups based on similar rates of hospital mortality (different colors in Tables 2 and 3 and Additional files 3 and 4: Tables S1 and S2). To rule out the fact that our findings are merely another way to explore the higher mortality rates in patients meeting both UO and sCr criteria for AKI [16], we performed a sensitivity analysis using only SCr-based KDIGO criteria against reduced KeGFR (Additional file 3: Table S1). Overall, the results did not change. For further comparison, we evaluated the eGFR using the CKD-EPI formula with the highest SCr. As shown in Additional file 5: Table S3, although there was an increment in mortality according to eGFR, this remained true only in patients with no AKI. There was no stepwise increment in mortality in each AKI severity stage 1 through 3 according to eGFR by CKD-EPI.

To further explore the association of the worst achieved KeGFR and hospital mortality, we adjusted this association for potential confounders and evaluated the adjusted odds ratio in each AKI stage (Table 4). Again, eGFR by CKD-EPI was independently associated with hospital mortality only in patients with no AKI (Additional file 6: Table S4).

**Table 4:** Adjusted odds ratios for hospital death

	Odds ratio (95% confidence interval)			
KeGFR (ml/min/1.73 m^2^)	No AKI (*n* = 3922)	AKI stage 1 (*n* = 3996)	AKI stage 2 (*n* = 3826)	AKI stage 3 (*n* = 1540)
>70	Reference	Reference	Reference	Reference
45–70	1.39 (1.00–1.94)	2.41 (1.91–3.19)	0.86 (0.66–1.32)	1.79 (1.09–2.54)
30–45	1.45 (0.64–2.986)	2.47 (1.44–4.35)	1.62 (1.28–1.71)	2.28 (1.38–2.63)
<30	3.71 (2.14–5.90)	5.43 (3.48–7.99)	1.73 (1.34–2.31)	4.39 (2.59–5.29)

Observe that the worst KeGFR is associated with hospital death even after dividing patients by maximum AKI stage

Adjusted for age, gender, SAPS II score, SOFA score, main comorbidities (hypertension, congestive heart failure, cardiac arrhythmias, chronic pulmonary obstructive disease, diabetes mellitus, lymphoma, metastatic cancer, liver disease, obesity), type of admission (clinical or surgical), baseline estimated glomerular filtration rate, need for vasoactive drugs, and mechanical ventilation

*AKI* acute kidney injury, *KeGFR* kinetic estimated glomerular filtration rate

### Worst KeGFR and need for RRT

We also evaluated if the worst KeGFR was associated with a need for RRT beyond the KDIGO system during ICU stay. In Table 2, we can observe that in the group of patients with KDIGO stage 3 and a worst achieved KeGFR greater than 30 ml/min/1.73 m^2^ or a worst achieved KeGFR < 30 ml/min/1.73 m^2^ and KDIGO stage 1/2, the rate of RRT was no greater than 10%. However, this rate was almost 44% when both KDIGO stage 3 and a worst KeGFR < 30 ml/min/1.73 m^2^ were observed.

### AKI, the worst achieved KeGFR, and long-term survival

**Fig. 2:**
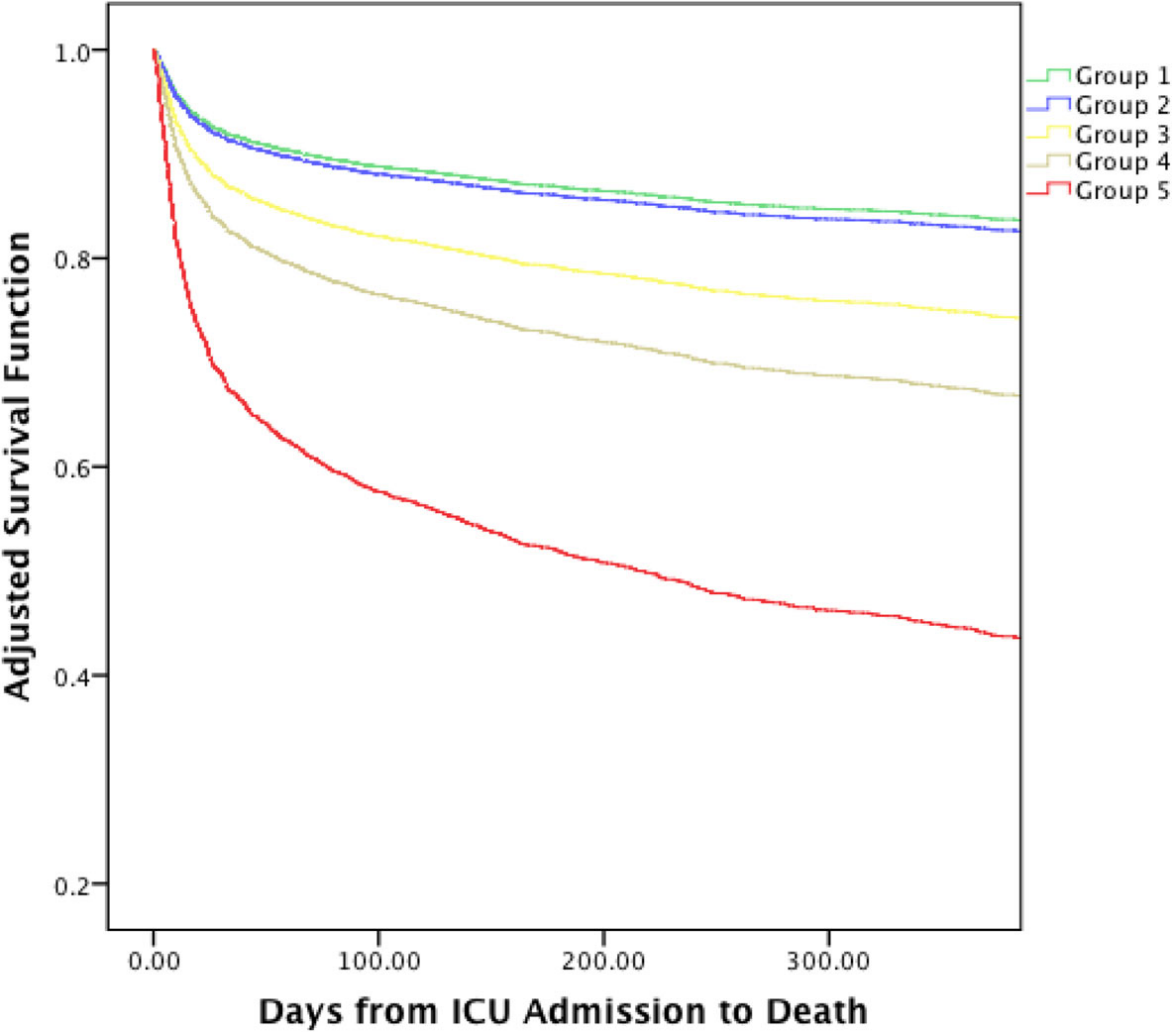
Comorbidities, baseline eGFR, and age-adjusted survival by AKI severity and worst KeGFR. Groups refer to combinations of maximum AKI stage and worst achieved KeGFR depicted in Table 4 and Additional file 4: Table S2. Group 1 (*green*), no AKI and KeGFR ≥ 30 ml/min/1.73 m^2^ or AKI stage 1 and KeGFR ≥ 70 ml/min/1.73 m^2^; group 2 (*blue*), AKI stage 1 and KeGFR between 30 and 70 ml/min/1.73 m^2^; group 3 (*yellow*), AKI stage 2 and KeGFR > 45 ml/min/1.73 m^2^ or AKI stage 3 and KeGFR ≥ 70 ml/min/1.73 m^2^ or no AKI/AKI stage 1 and KeGFR < 30 ml/min/1.73 m^2^; group 4 (*brown*), AKI stage 2 and KeGFR < 45 ml/min/1.73 m^2^ or AKI stage 3 and KeGFR between 30 and 69 ml/min/1.73 m^2^; group 5 (*red*), AKI stage 3 and KeGFR < 30 ml/min/1.73 m^2^. The top panel shows age-adjusted 1-year survival for all patients (10 patients had missing age). Overall differences per groups were significant (*p* < 0.001). *ICU* intensive care unit

After adjusting for comorbidities, baseline eGFR, and age, survival over 1 year after ICU admission followed a similar pattern to the hospital death shown in Table 2. In Fig. 2, there was separation among all, except one, of the five groups depicted in Table 3.

### Sensitivity analysis

Besides a separated analysis considering only patients with ICU admission eGFR > 70 ml/min/1.73 m^2^ and considering only sCr-based KDIGO criteria, we performed another subgroup analysis with non-oliguric patients only (KDIGO stage 3 according to urine criterion were excluded) and the results are shown in Additional file 3: Table S1. In general, the main results were maintained regarding the need for RRT and hospital mortality.

## Discussion

In this study, the performance of KeGFR in critically ill patients was evaluated for the first time. We found that the worst achieved KeGFR within the first 7 days of ICU stay was associated with several short- and long-term outcomes, such as the need for RRT, hospital mortality, and 1-year survival. Moreover, the worst KeGFR appears not to substitute for, but adds prognostic information to the current AKI classification.

Although significant advances have been made in the diagnosis and prognosis of AKI since the development of the consensus classification system, several questions remained when evaluating patients in this setting. First, as stated in the introduction section, current AKI classifications are not able to discern prognosis between patients with pure AKI or acute-on-chronic kidney disease [8]. Another possible pitfall concerns the time patients take to fully develop AKI severity, as exemplified in the introduction section when one patient had an increment of 50% of baseline SCr within 12 h and another within 48 h, but both were classified as KDIGO stage 1. Theoretically, calculating KeGFR even when SCr changes acutely can avoid these gaps found in the AKI classification system.

Our results demonstrate several important findings. First, we disclosed a disagreement between AKI severity and the worst achieved KeGFR. Several patients had AKI KDIGO stage 3, but maintained KeGFR greater than 70 ml/min/1.73 m^2^. This can be explained by a slow increment of SCr over time. For example, one patient had a baseline SCr of 0.6 mg/dl and it increased only approximately 0.3 mg/dl each 48 h, going up to 1.8 mg/dl after 7 days. This patient was classified as AKI stage 3, but his KeGFR was never lower than 70 ml/min/1.73 m^2^. On the other hand, other patients had severely reduced KeGFR but no or only a minor AKI stage. Clearly, some of these patients already had reduced eGFR at baseline. However, when evaluating only those patients admitted with eGFR above 70 ml/min/1.73 m^2^ (Table 3) we can identify that most of these patients had no eGRF reduction at baseline. In these cases, the increase in SCr was not so great, but occurred within a short time interval (for example, an increment of 0.3 mg/dl in two consecutive SCr measurements, obtained 8 h apart can reduce the KeGFR to less than 30 ml/min/1.73 m^2^, but this patient will be classified as only AKI stage 1).

Although the great majority of SCr measurements in the present study had an interval between them of 20 to 28 h, we maintained all measurements with an interval between 6 and 48 h, making it possible to evaluate the patients earlier, within the first 12 h after ICU admission, when SCr can already be increasing. At this time, it is possible there is not enough time for SCr to increase for the KDIGO system to achieve even AKI stage 1, although KeGFR can already be severely reduced.

Secondly, and perhaps most importantly, both the AKI classification system and KeGFR seem to be complementary in predicting outcomes. For example, almost 45% of patients with AKI stage 3 and KeGFR < 30 ml/min/1.73 m^2^ needed RRT in comparison with less than 10% of patients with AKI stage 3 but less severe KeGFR reduction and less than 5% of those patients with KeGFR < 30 ml/min/1.73 m^2^ but only AKI stage 1/2.

In relation to hospital mortality, a stepwise reduction in the worst achieved KeGFR conferred an incremental risk of death to each AKI stage in both uni- and multivariate analyses. It is already known that AKI classification systems are not as good at predicting events in patients with previous CKD [7, 8]. Moreover, it has been recently suggested that different AKI patterns in relation to SCr trajectory (resolving/nonresolving) imply different prognoses [17]. Analyzing the KeGFR equation, it contains two important pieces of information not contemplated in the AKI classification systems: baseline eGFR and the speed of SCr increase. In part, it is probable that KeGFR adds prognostic information because it can identify patients with previous CKD. However, our data suggest that to correctly quantify renal injury in critically ill patients we must take into consideration not only the SCr increment degree but also the speed at which this increment occurs, as suggested in the introductory section of this manuscript. Supported by the groups shown in Table 3, it is important to highlight that we do not propose substituting the AKI classification with KeGFR, but we think both must be used together—the first to evaluate the magnitude of the acute injury and the latter to measure the effects of AKI on GFR.

Because it is clear that oligoanuric patients had GFR close to zero and it is more difficult to ascertain eGFR in these patients who maintain UO, we performed a sensitivity analysis excluding those patients with AKI KDIGO stage 3 according to the UO. Generally, the results were maintained, mainly when evaluating the need for RRT (almost 50% of patients with both AKI stage 3 according to the Scr criterion and a KeGFR < 30 ml/min/1.73 m^2^).

Finally, we also evaluated long-term mortality. Except for groups 1 and 2, there was a clear separation in survival lines according to the classification by AKI KDIGO stage/worst KeGFR. These results emphasize that AKI severity alone does not determine long-term outcome but that an interaction between baseline GFR and AKI severity and the speed of onset of AKI are important to assess both short- and long-term prognosis.

Our study has several and important limitations. First, and most important, we did not have access to previous SCr measurements thus making it impossible to identify patients with actual previous CKD. To overcome this fact, we performed a sensitivity analysis including only patients with an eGFR > 70 ml/min/1.73 m^2^. We considered the lowest SCr available during ICU stay as the baseline, although this approach can inflate the AKI incidence, indicating that such a level is often lower than the most recent outpatient creatinine value [18]. Another limitation is that it is difficult to determine the actual maximal increase in SCr when eGFR is near zero in critically ill patients and, consequently, the total body water volume as described in the methodology section. Although it has been suggested to limit this increment to 1.7 mg/dl a day [9, 10], we analyzed a subset of patients with anuria and no RRT to determine the mean value of daily SCr increment (a real measure of maximal SCr increment) and used different means for men and women, although we acknowledge this can change according to obesity status, age, and other factors. While we have identified KeGFR as a prognostic tool in risk stratification regarding the need for RRT and survival, identifying patients at high risk and highlighting the importance of implementing measures that prevent/limits further renal damage [19], we recognize that, regarding the further practical use of KeGFR for drug dose correction, validation studies using standard GFR measurements (by measuring CrCl or using exogenous substances such as inulin, iohexol, and others) are warranted.

## Conclusion

In conclusion, we suggest that both the AKI classification system and KeGFR are complementary to each other. Analyzing different prognoses according to the worst achieved KeGFR in each AKI stage, we suggest that patients with acute CKD, pure AKI, slow- or fast-onset AKI, and all possible combinations thereof, have different prognoses and that assessing both AKI stage and KeGFR can help to identify patients at different risk levels in clinical practice.

## Additional files

Additional file 1: Illustrative cases of differences between KDIGO stage and worst KeGFR. (DOCX 14 kb)

Additional file 2: Figure S1. Discriminative ability of worst KeGFR as continuous or categorized variable in predicting hospital death. (DOCX 36 kb)

Additional file 3: Table S1. Outcomes for patients according to maximum sCr-based AKI severity and worst achieved eGFR. (DOCX 16 kb)

Additional file 4: Table S2. Outcomes for non-oliguric patients according to maximum sCr-based AKI severity and worst achieved eGFR. (DOCX 16 kb)

Additional file 5: Table S3. Outcomes for patients according to maximum sCr-based AKI severity and worst eGFR estimated by CKD-EPI equation using maximum SCr. (DOCX 16 kb)

Additional file 6: Table S4. Adjusted odds ratios for hospital death. Observe that worst eGFR estimated by CKD-EPI equation using maximum SCr is associated with hospital death only in patients with no AKI. (DOCX 15 kb)

## Abbreviations

AKI: Acute kidney injury
CKD: Chronic kidney disease
CrCl: Creatinine clearance
ESRD: End-stage renal disease
GFR: Glomerular filtration rate
ICU: Intensive care unit
KDIGO: Kidney Disease Improving Global Outcomes
KeGFR: Kinetic estimated glomerular filtration rate
LOS: Length of stay
MIMIC: Multiparameter Intelligent Monitoring in Intensive Care
RRT: Renal replacement therapy
SAPS: Simplified Acute Physiology Score
SCr: Serum creatinine
SOFA: Sequential Organ Failure Assessment
UO: Urine output
