# Supplementary material for: Kinetic estimated glomerular filtration rate in critically ill patients: beyond the acute kidney injury severity classification system
Source: Crit Care. 2017 Nov 18;21:280. doi: 10.1186/s13054-017-1873-0 (PMC5694169; doi:10.1186/s13054-017-1873-0)
Supplement: Supplementary file 1 — Illustrative cases of differences between KDIGO stage and worst KeGFR. (DOCX 14 kb) [file 13054_2017_1873_MOESM1_ESM.docx]

**Additional file 1**

**Illustrative Case**

**Case 1: Consider a male,30 years-old and black in 2 distinct situations.**

|  | **Situation 1** | **Situation 2** |
| --- | --- | --- |
| **Time 0** | **SCr: 1 mg/dL** | **SCr: 1 mg/dL** |
| **After 12h** | **SCr: 1.5 mg/dL** | **SCr: 1.2 mg/dL** |
| **After 48h** | **SCr: 1.5 mg/dL** | **SCr: 1.5 mg/dL** |
| **After 72h** | **SCr: 1.5 mg/dL** | **SCr: 1.5 mg/dL** |
| **AKI stage** | **KDIGO 1** | **KDIGO 1** |
| **Baseline eGFR (CKD-EPI)** | **116ml/min/1.73m^2^** | **116ml/min/1.73m^2^** |
| **KeGFR at time 0** | **116ml/min/1.73m^2^** | **116ml/min/1.73m^2^** |
| **KeGFR after 12h** | **29.8ml/min/1.73 m^2^** | **77.1 ml/min/1.73m^2^** |
| **KeGFR after 48h** | **77.7 ml/min/1.73m^2^** | **74.6 ml/min/1.73m^2^** |
| **KeGFR after 72h** | **77.7 ml/min/1.73m^2^** | **77.7 ml/min/1.73m^2^** |

**Patients had the same KDIGO classification, but different worst KeGFR – shadow.**

**Case 2: Consider a female, 55 years-old, non-black in 2 distinct situations.**

|  | **Situation 1** | **Situation 2** |
| --- | --- | --- |
| **Time 0** | **SCr: 0.8 mg/dL** | **SCr: 2 mg/dL** |
| **After 12h** | **SCr: 0.9 mg/dL** | **SCr: 2.2 mg/dL** |
| **After 48h** | **SCr: 1.0 mg/dL** | **SCr: 2.5 mg/dL** |
| **After 72h** | **SCr: 1.2 mg/dL** | **SCr: 3.0 mg/dL** |
| **AKI stage** | **KDIGO 1** | **KDIGO 1** |
| **Baseline eGFR (CKD-EPI)** | **83ml/min/1.73m^2^** | **27.4ml/min/1.73m^2^** |
| **KeGFR at time 0** | **83ml/min/1.73m^2^** | **27.4ml/min/1.73m^2^** |
| **KeGFR after 12h** | **67.0ml/min/1.73 m^2^** | **18.7 ml/min/1.73m^2^** |
| **KeGFR after 48h** | **66.5 ml/min/1.73m^2^** | **20.0 ml/min/1.73m^2^** |
| **KeGFR after 72h** | **51.8 ml/min/1.73m^2^** | **12.8ml/min/1.73m^2^** |

**Shadow: Worst KeGFR.**

**Patients had the same KDIGO classification, but different worst KeGFR - shadow.**
