# Supplementary material for: Kinetic estimated glomerular filtration rate in critically ill patients: beyond the acute kidney injury severity classification system
Source: Crit Care. 2017 Nov 18;21:280. doi: 10.1186/s13054-017-1873-0 (PMC5694169; doi:10.1186/s13054-017-1873-0)

**Additional file 2: Figure S1:** Discriminative ability of worst KeGFR as continuous or categorized variable in predicting hospital death.


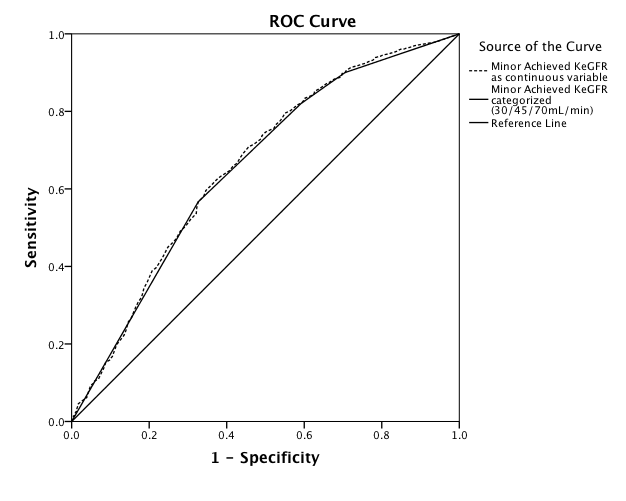

Supplement: Supplementary file 2 — Discriminative ability of worst KeGFR as continuous or categorized variable in predicting hospital death. (DOCX 36 kb) [file 13054_2017_1873_MOESM2_ESM.docx]
