# Supplementary material for: Kinetic estimated glomerular filtration rate in critically ill patients: beyond the acute kidney injury severity classification system
Source: Crit Care. 2017 Nov 18;21:280. doi: 10.1186/s13054-017-1873-0 (PMC5694169; doi:10.1186/s13054-017-1873-0)
Supplement: Supplementary file 3 — Outcomes for patients according to maximum sCr-based AKI severity and worst achieved eGFR. (DOCX 16 kb) [file 13054_2017_1873_MOESM3_ESM.docx]

|  | **Worst Achieved KeGFR during ICU stay** | | | | |
| --- | --- | --- | --- | --- | --- |
| **KDIGO stage**  **Creatinine-based** | **>70mL/min** | **45-70mL/min** | **30-45mL/min** | **<30mL/min** | **Total** |
| **No AKI**  **Dead (%)**  **RRT (n,%)** | 6,081  6.9  0.5 | 2,163  10.1  0.8 | 424  11.6  2.4 | 396  16.2  0.5 | 9,064  8.6  0.6 |
| **Stage 1**  **Dead (%)**  **RRT (n,%)** | 412  8.4  0.7 | 663  13.0  1.1 | 265  12.1  1.1 | 251  26.  2.8 | 1,591  13.8  1.3 |
| **Stage 2**  **Dead (%)**  **RRT (n, %)** | 384  7.5  - | 398  12.8  0.8 | 260  19.6  6.2 | 385  28.0  10.9 | 1,427  16.7  4.3 |
| **Stage 3**  **Dead (%)**  **RRT (n, %)** | 212  9.0  8.9 | 196  17.3  8.7 | 163  29.4  13.5 | 631  35.5  47.2 | 1,202  26.9  29.6 |
| **Total**  **Dead (%)**  **RRT (%)** | 7,089  7.0  0.7 | 3,420  12.2  1.3 | 1,112  16.2  4.6 | 1,663  27.8  21.0 | 13,284  11.8  3.7 |

**Additional file 3: Table S1:** Outcomes for patients according to maximum sCr-based AKI severity and worst achieved KeGFR.

RRT: renal replacement therapy
