# Supplementary material for: Kinetic estimated glomerular filtration rate in critically ill patients: beyond the acute kidney injury severity classification system
Source: Crit Care. 2017 Nov 18;21:280. doi: 10.1186/s13054-017-1873-0 (PMC5694169; doi:10.1186/s13054-017-1873-0)
Supplement: Supplementary file 4 — Outcomes for non-oliguric patients according to maximum sCr-based AKI severity and worst achieved eGFR. (DOCX 16 kb) [file 13054_2017_1873_MOESM4_ESM.docx]

**Additional file 4: Table S2:** Outcomes for non-oliguric patients according to maximum sCr-based AKI severity and worst achieved KeGFR.

|  | **Worst Achieved KeGFR during ICU stay** | | | | |
| --- | --- | --- | --- | --- | --- |
| **KDIGO stage**  **Urinary and sCr-based** | **>70mL/min** | **45-70mL/min** | **30-45mL/min** | **<30mL/min** | **Total** |
| **No AKI**  **Dead (%)**  **RRT (%)** | 2,764  4.1  0.3 | 841  6.4  0.4 | 143  6.3  - | 174  12.1  0.6 | 3,922  5.0  0.3 |
| **Stage 1**  **Dead (%)**  **RRT (%)** | 2,322  4.0  0.5 | 1,124  9.5  1.4 | 304  9.5  1.6 | 246  15.0  1.6 | 3,996  6.6  0.9 |
| **Stage 2**  **Dead (%)**  **RRT (%)** | 1,689  14.3  0.4 | 1,169  16.1  0.4 | 457  19.9  6.3 | 511  22.1  4.3 | 3,826  16.5  1.6 |
| **Stage 3**  **Dead (%)**  **RRT (%)** | 222  3.6  6.8 | 189  8.5  9.0 | 146  15.8  9.6 | 336  29.8  38.7 | 888  15.8  19.7 |
| **Total**  **Dead (%)**  **RRT (%)** | 6,997  6.6  0.6 | 3,323  11.8  1.2 | 1,050  14.2  4.1 | 1,267  21.9  12.7 | 12,637  9.8  2.2 |

RRT: renal replacement therapy
