# Supplementary material for: Kinetic estimated glomerular filtration rate in critically ill patients: beyond the acute kidney injury severity classification system
Source: Crit Care. 2017 Nov 18;21:280. doi: 10.1186/s13054-017-1873-0 (PMC5694169; doi:10.1186/s13054-017-1873-0)
Supplement: Supplementary file 5 — Outcomes for patients according to maximum sCr-based AKI severity and worst eGFR estimated by CKD-EPI equation using maximum SCr. (DOCX 16 kb) [file 13054_2017_1873_MOESM5_ESM.docx]

**Additional file 5: Table S3:** Outcomes for patients according to maximum sCr-based AKI severity and worst eGFR estimated by CKD-EPI equation using maximum SCr.

|  | **Worst eGFR using CKD-EPI equation during ICU stay** | | | | |
| --- | --- | --- | --- | --- | --- |
| **KDIGO stage**  **Urine and sCr-based** | **>70mL/min** | **45-70mL/min** | **30-45mL/min** | **<30mL/min** | **Total** |
| **No AKI**  **Dead (%)**  **RRT (%)** | 2,350  4.3  0.6 | 1,412  5.9  0.3 | 77  6.5  1.3 | 83  8.4  1.2 | 3,922  5.0  0.3 |
| **Stage 1**  **Dead (%)**  **RRT (%)** | 1,570  5.2  0.5 | 1,357  6.1  1.3 | 1,044  9.4  1.1 | 25  8.0  - | 3,996  6.6  0.9 |
| **Stage 2**  **Dead (%)**  **RRT (%)** | 869  17.4  0.8 | 1,084  18.1  0.7 | 786  18.9  1.4 | 1,087  12.4  3.2 | 3,826  16.5  1.6 |
| **Stage 3**  **Dead (%)**  **RRT (%)** | 49  59.1  4.1 | 97  49.4  4.1 | 83  59.0  7.2 | 1,311  26.0  28.4 | 1,540  30.4  25.0 |
| **Total**  **Dead (%)**  **RRT (%)** | 4,838  7.5  0.5 | 3,950  10.4  0.9 | 1,990  15.1  1.5 | 2,506  19.4  16.3 | 13,284  11.8  3.7 |

RRT: renal replacement therapy; eGFR: estimated glomerular filtration rate; SCr: serum creatinine.
