# Supplementary material for: Kinetic estimated glomerular filtration rate in critically ill patients: beyond the acute kidney injury severity classification system
Source: Crit Care. 2017 Nov 18;21:280. doi: 10.1186/s13054-017-1873-0 (PMC5694169; doi:10.1186/s13054-017-1873-0)
Supplement: Supplementary file 6 — Adjusted odds ratios for hospital death. Observe that worst eGFR estimated by CKD-EPI equation using maximum SCr is associated with hospital death only in patients with no AKI. (DOCX 15 kb) [file 13054_2017_1873_MOESM6_ESM.docx]

|  | No AKI  (n=3,922) | AKI stage 1  (n=3,996) | AKI stage 2  (n=3,826) | AKI stage 3  (n=1,540) |
| --- | --- | --- | --- | --- |
|  | OR (95%CI) | OR (95%CI) | OR (95%CI) | OR (95%CI) |
| KeGFR  >70mL/min  45-70mL/min  30-45mL/min  <30mL/min | Reference  1.34 (0.76-2.39)  3.12 (1.96-7.66)  4.0 0(2.11- 10.14) | Reference  0.82 (0.59-1.12)  0.87 (0.58-1.31)  1.41 (0.85-2.35) | Reference  0.78 (0.60-1.08)  0.81 (0.61-1.06)  0.79 (0.58-1.08) | Reference  1.07 (0.68-1.66)  0.84 (0.56-1.76)  0.77 (0.52-1.14) |

**Additional file 6: Table S4:** Adjusted odds ratios for hospital death. Observe that worst eGFR estimated by CKD-EPI equation using maximum SCr is associated with hospital death only in patines with no-AKI.

Adjusted for age, gender, simplified acute physiology score II (SAPS-II), sequential organ failure assessment (SOFA), main comorbidities (hypertension, congestive heart failure, cardiac arrhythmias, chronic pulmonary obstructive disease, diabetes mellitus, lymphoma, metastatic cancer, liver disease, obesity), type of admission (clinical or surgical), baseline estimated glomerular filtration rate, need for vasoactive drugs and mechanical ventilation
